# Serum and Urine α — Amylase Isoenzymes levels After Operative Cholangiogram

**DOI:** 10.1155/1990/92548

**Published:** 1990

**Authors:** Evaghelos Xynos, Evaghelos Neonakis, George Pechlivanidis, John S. Vassilakis

**Affiliations:** 1 Second Surgical Department Athens Naval and Veterans Hospital and the Surgical Unit Medical School University of Crete Greece; 2 49 Ymittou Str. Cholargos Athens GR-155 61 Greece

## Abstract

Serum and urine total α-amylase isoenzymes values were estimated in two groups of patients, who underwent either elective cholecystectomy and operative cholangiogram (group A — 59 patients) or cholecystectomy without operative cholangiogram (group B — 68 patients). Serum and urine total α-amylase and pancreatic isoamylase (p-type) values were statistically significantly increased within the first 24 postoperative hours as compared to the preoperative levels only in group A (p < 0.05). No clinical signs of pancreatitis were observed. Serum lipase alterations did not reach any statistically significant difference in either group. It is concluded that transient hyperamylasaemia after peroperative cholangiogram may be due to a reversible chemical pancreatitis caused by the infused opacifying agent into the common bile duct.


*HPB Surgery*, 1990, Vol. 3, pp. 47–52
Reprints available directly from the publisher
Photocopying permitted by license only

© 1990 Harwood Academic Publishers GmbH
Printed in the United Kingdom

### SERUM AND URINE $\alpha$ — AMYLASE ISOENZYMES LEVELS AFTER OPERATIVE CHOLANGIOGRAM

### A Prospective Clinical and Biochemical Study

EVAGHELOS XYNOS*, EVAGHELOS NEONAKIS, GEORGE PECHLIVANIDIS and JOHN S. VASSILAKIS
*Second Surgical Department, Athens Naval and Veterans Hospital and the Surgical Unit, Medical School, University of Crete.*

*(Received 7 November 1989)*

Serum and urine total $\alpha$-amylase isoenzymes values were estimated in two groups of patients, who underwent either elective cholecystectomy and operative cholangiogram (group A — 59 patients) or cholecystectomy without operative cholangiogram (group B — 68 patients). Serum and urine total $\alpha$-amylase and pancreatic isoamylase (p-type) values were statistically significantly increased within the first 24 postoperative hours as compared to the preoperative levels only in group A ($p < 0.05$). No clinical signs of pancreatitis were observed. Serum lipase alterations did not reach any statistically significant difference in either group. It is concluded that transient hyperamylasaemia after peroperative cholangiogram may be due to a reversible chemical pancreatitis caused by the infused opacifying agent into the common bile duct.

KEY WORDS: Cholangiogram, cholecystectomy, amylase, lipase, pancreatitis

### INTRODUCTION

Transient hyperamylasaemia of 9–32 percent following abdominal operations as well as a non significant increase in total serum and urine $\alpha$-amylase levels following peroperative cholangiogram have been reported[1–6].

In the present study serum and urine total $\alpha$-amylase, pancreatic isoamylase (p-type), salivary isoamylase (s-type) and serum lipase levels after peroperative cholangiogram were determined in a prospective double controlled study.

### PATIENTS AND METHODS

One hundred and twenty seven patients were randomly divided into two groups. Fifty nine patients underwent elective cholecystectomy with peroperative cholangiogram (group A) and another 68 patients underwent elective cholecystectomy without peroperative cholangiogram (group B). Their ages ranged between 38 and 72 years (mean: 57) in group A and between 35 and 76 (mean: 55) in group B.

Address for correspondence: Evaghelos Xynos, 49 Ymittou Str., Cholargos, Athens, GR–155 61, Greece.

Patients with acute pancreatitis, acute cholecystitis and choledocholithiasis or negative exploration of the common bile duct, as well as with a positive history of pancreatic, renal or liver disease were excluded from the study. Two films were taken in all patients who had peroperative cholangiogram with a total infusion of 8–10 ml of adipiodon methyl glycamine (Biligraphin) 25 percent solution into the common bile duct through the cystic duct.

Serum and urine total $\alpha$-amylase, p-type isoamylase and s-type isoamylase levels were measured before, during (15 min after the cholangiogram) and 6, 12 and 24 hours after the operation. Serum lipase levels were measured 24 hours before and 24 hours after the operation. Pancreatic and salivary type $\alpha$-amylase isoenzymes estimations were performed using the chromogenic enzyme test (Rhadebas R Isoamylase Test, Pharmacia Diagnostics). Student-t-test for paired values was applied in the statistical analysis of the results.

### RESULTS

Mean serum total $\alpha$-amylase and p-type isoamylase levels 6 hours after the operation were found to be statistically significantly increased as compared to the respective levels before operation in group A ($p < 0.05$ and $p < 0.01$ respectively) (Table 1). Furthermore urine total $\alpha$-amylase and p-type isoamylase levels 12 and 24 hours postoperatively were found to be statistically significantly increased in comparison with the respective values before the operation ($p < 0.05$ and $p < 0.01$ respectively) (Table 1).

The differences between preoperative and postoperative serum and urine total $\alpha$-amylase, p-type isoamylase and s-type isoamylase levels in all time points in group B (Table 2), as well as between pre- and postoperative serum lipase in both groups were insignificant (group A: preop: 0,9 ± 0.2SD Units/mL, postop: 1.2 ± 0.3SD Units/mL and group B: preop: 0.8 ± 0.2SD Units/mL, postop: 1.2 ± 0.5SD Units/mL).

**Table 1** Group A serum and urine $\alpha$-amylase and p- and s-type isoamylase values (mean ± SD Units/mL).

| | total | p-type | s-type | p/s |
|---|---|---|---|---|
| | | SERUM | | |
| pre-op | 4.0±0.9 | 2.2±0.33 | 1.8±0.3 | 1.20 |
| per-op | 3.6±1.0 | 2.1±0.25 | 1.5±0.2 | 1.40 |
| post-op 6 hours | 5.0±1.6* | 3.3±0.9** | 1.7±0.5 | 1.90 |
| 12 hours | 3.9±1.1 | 2.5±0.6 | 1.4±0.3 | 1.80 |
| 24 hours | 4.0±1.1 | 2.6±0.6 | 1.4±0.3 | 1.80 |
| | | URINE | | |
| pre-op | 12.1±3.4 | 8.5±3.0 | 3.6±2.1 | 2.40 |
| post-op 6 hours | 12.6±3.7 | 9.7±3.9 | 2.9±1.4 | 3.30 |
| 12 hours | 17.1±4.7* | 13.3±4.8** | 3.8±2.8 | 3.50 |
| 24 hours | 16.7±5.0* | 12.8±4.9* | 3.9±3.0 | 3.30 |

*p<0.05, **p<0.01

**Table 2** Group B serum and urine $\alpha$-amylase and p- and s-type isoamylase values (mean ± SD Units/mL).

| | total | p-type | s-type | p/s |
|---|---|---|---|---|
| | | SERUM | | |
| pre-op | 4.3±0.7 | 2.2±0.37 | 2.1±0.21 | 1.05 |
| per-op | 4.3±0.9 | 2.5±0.41 | 1.8±0.31 | 1.40 |
| post-op 6 hours | 4.1±0.9 | 2.1±0.6 | 2.0±0.35 | 1.05 |
| 12 hours | 3.7±0.77 | 1.9±0.6 | 1.8±0.55 | 1.05 |
| 24 hours | 4.2±1.0 | 2.3±0.4 | 1.9±0.27 | 1.20 |
| | | URINE | | |
| pre-op | 11.8±4.1 | 8.3±2.8 | 3.5±1.2 | 2.4 |
| post-op 6 hours | 13.7±5.7 | 9.4±2.6 | 4.4±0.9 | 2.1 |
| 12 hours | 14.0±4.2 | 9.1±3.1 | 3.9±2.0 | 2.3 |
| 24 hours | 10.9±3.7 | 7.7±2.4 | 3.2±1.6 | 2.4 |

Opacification of the pancreatic duct on peroperative cholangiogram was evident in four cases of group A (7 percent). The postoperative increase in serum total $\alpha$-amylase and p-type isoamylase in those four patients was within the same range of values observed in the remaining cases of the same group.

No clinical signs of acute pancreatitis were observed in any patient of either group.

### DISCUSSION

Transient hyperamylasaemia following abdominal surgery has been reported in a variety of percentages ranging between 9 and 32 percent[3–5,7,8,9]. Hyperamylasaemia with clinical manifestation of pancreatitis has been reported in four percent following cholecystectomy with or without common bile duct exploration in 840 cases[1]. On the other hand Vernava *et al.*, had no postoperative pancreatitis after intraoperative cholangiogram in their series[10]. It has been reported that abdominal surgery results in an increase in serum p-type isoamylase and s-type isoamylase in a ratio of 3:1[3,6]. Berdenheier *et al.*[1] report 8.7 and 4.7 percent increases in serum total amylase following cholecystectomy with operative cholangiogram and cholecystectomy alone respectively. On the contrary, Fahra *et al.*[2] report no serum total amylase level alterations following cholecystectomy with or without peroperative cholangiogram.

In the present study serum and urine amylase isoenzyme measurements at consecutive time points following cholecystectomy with or without cholecystectomy, as well as serum lipase measurements were performed. Since salivary isoamylase levels might have been increased following operation for several reasons (ie. manipulations during endotracheal intubation), amylase isoenzymes evaluation was considered essential in order to eliminate the contribution of salivary isoenzyme to a possible total $\alpha$-amylase alterations, assessing therefore exclusively pancreatic isoamylase changes induced by the peroperative cholangiogram. The results revealed a significant increase in serum total $\alpha$-amylase and ptype isoamylase 6 hours after cholecystectomy and peroperative cholangiogram in percentages of 25 and 50 respectively, and a significant increase in urine total $\alpha$-amylase and p-type isoamylase 12 and 24 hours after cholecystectomy and peroperative cholangiogram in percentages of 40 and 50 percent respectively. Those increases seem to be due exclusively to peroperative cholangiogram, since no significant respective changes were observed after cholecystectomy without peroperative cholangiogram.

The mechanism by which peroperative cholangiogram induces pancreatic hyperamylasaemia is unclear. The contemplation that the opacifying agent might reflux into the pancreatic duct and exert a direct toxic action on acinar cells is not supported by adequate evidence, since opacification of the pancreatic duct was radiologically confirmed in only four patients (7 percent) in group A. In support however of that hypothesis is the fact, that there is a strong correlation between the maximal increase in serum pancreatic enzymes and the degree of opacification of the pancreatic ductal system, at least after endoscopic retrograde pancreatography[11]. An alternative speculation might be, that peroperative cholangiogram may induce a transient spasm of the sphincter of Oddi causing bile and pancreatic juice to reflux into the pancreatic duct. Finally amylase release from pancreatic acinar cells might be the indirect result of a stimulation of lymphatic flow. Whatever the mechanism might be, there have been no signs of clinical manifestation of acute pancreatitis in either group of the present study and peroperative cholangiogram can be considered as a safe diagnostic procedure with negligible risks, as several authors have already confirmed[10,12]. Therefore serial postoperative determination of amylase levels appears to be useless.

### *References*

1. Bardenheier, J.A., Kaminski, D.L., William, V.L. (1968) Pancreatitis after biliary tract surgery. *Am. J. Surg.*, **116**, 773–6
2. Farha, G.J., Ammar, A.D., Chang, F.C. (1980) The incidence and significance of elevations of serum and urinary amylase levels following trancystic duct cholangiography. *Surg. Gynecol. Obstet.*, **151**, 769–72
3. Harada, K., Kitamura, M., Ikenaga, T. (1974) Isoenzyme study of postoperative transient hyperamylasemia. *Am. J. Gastroenterol.*, **61**, 121–6
4. Keighly, M.R.B., Johnson, A.G., Stevens, A.E. (1969) Raised serum amylase after upper abdominal operation. *Br. J. Surg.*, **56**, 424–7
5. Mahaffey, J.H., Howard, J.M. (1955) The incidence of postoperative pancreatitis: Study of 131 surgical patients utilizing serum amylase concentration. *Arch. Surg.*, **70**, 348–52
6. Morrissey, R., Berk, E., Fridhandler, L., Daniel, P. (1974) The nature and significance of hyperamylasemia following operation. *Ann. Surg.*, **180**, 67–71
7. Perryman, R.G., Hoer, S.O. (1954) Observation on postoperative pancreatitis and postoperative elevation of serum amylase. *Am. J. Surg.*, **88**, 417–20
8. Singh, L.M., Okukubo, F., James, P.M., Salmon, J., Howarf, J.M. (1965) Further studies on postoperative pancreatitis. *Arch. Surg.*, **90**, 43–9
9. Miller, S.F., Whitaker, J.R. Jr., Snyder, R.D. (1973) Incidence of elevated serum amylase levels and pancreatitis after upper abdominal surgery. *Am. J. Surg.*, **125**, 535–7
10. Vernava, A., Andrus, C., Herrmann, V.M., Kaminski, D.L. (1987) Pancreatitis after biliary tract surgery. *Arch. Surg.*, **122**, 575–80
11. Okuno, M., Himeno, S., Kurokawa, M., Shinomura, Y., Kuroshima, T., Kanayama, S., Tsuji, K., Higashimoto, Y., Tarui, S. (1985) Changes in serum levels of pancreatic isoamylase, lipase, trypsin and elastase 1 after endoscopic retrograde pancreatography. *Hepatogastroenterology*, **32**, 87–90

12. Kitahama, A., Kerstein, M.D., Overby, J.L., Kappelman, M.D., Webb, W.R. (1986) Routine intraoperative cholangiogram. *Surg. Gynecol. Obstet.*, **162**, 317–22

*(Accepted by S. Bengmark on 8th November 1989)*

### INVITED COMMENTARY

The authors have made a study on the possible danger of doing per-operative cholangiography during elective cholecystectomy. They find a small increase in serum and urine total as well as pancreatic isoamylase (p-type) within the first 24 postoperative hours, but no clinical signs of pancreatitis in any patient. Their sound conclusion is, that "peroperative cholangiogram can be considered as a safe diagnostic procedure with negligible risks". I think this indeed is important, since per-operative cholangiography is a prerequisite to keep the number of patients with retained postoperative biliary stones at a minimum.

I really wonder if the small increase in pancreatic amylase is a sign of chemical pancreatitis? Such a small increase in amylase is fairly non-specific and can be seen in a variety of different diseases without pancreatitis, as well as after various surgical procedures well outside the pancreas, as also pointed out by the authors. The use of a more specific test for acute pancreatitis, as e.g. trypsin-alpha-I-protease inhibitor complexes in plasma, could be useful to answer this question. When we compare amylase and trypsin-alpha-1-protease inhibitor complexes after diagnostic ERCP we could see that about 40% of the patients had a raised serum amylase level after the investigation compared with only about 10% showing raised complex levels (unpubl. results). The latter correlates much better than amylase with clinical symptoms, such as abdominal pain or overt acute pancreatitis.

The contrast medium used might also explain their findings in many different ways. Biligraphin is a quite toxic substance, and is nowadays usually replaced by less toxic contrast media. It is known that Biligraphin could cause transient rise in liver enzyme levels, especially in patients taking oral contraceptives. Could this explain the findings? Did the authors make any comparison with liver enzyme levels? If so, was there any correlation? Were there perhaps more women on oral contraceptives in the group undergoing cholangiography than in the "control" group?

The contrast medium could perhaps also alter the intestinal permeability within the duodenum by way of its high viscosity and osmolarity? This might give increased reabsorbtion of pancreatic enzymes, as e.g. amylase, to plasma, in agreement with our results seen in patients with acute duodenitis. When studying the diagnostic value of raised plasma levels of trypsin-alpha-1-protease inhibitor complexes in acute pancreatitis, we found five patients with duodenitis at acute gastroscopy as the only explanation to rather high complex levels in plasma. The same pattern was seen in another four patients with a perforated duodenal ulcer in this study. These two groups of patients, together with patients with acute pancreatitis, were the only ones with raised complex levels among 220 patients presenting with acute abdominal pain at our Surgical Emergency Unit during a one month period.

Otherwise, the authors suggestion about reflux of contrast medium into the pancreatic duct as a possible cause of the raised amylase levels seems quite logical, especially bearing the toxicity of Biligraphin in mind. Earlier studies have shown that 60% of the patients who get successful opacification of the pancreas at ERCP have raised amylase levels after the ERCP. This is also in line with the concept of overfilling of the pancreas as the cause of acute pancreatitis, which occurs as a complication in about 1% after diagnostic ERCP. The possibility of a transient spasm of the sphincter of Oddi seems also quite probable to give raised amylase levels without causing pancreatitis.

To conclude, using as non-toxic contrast media as possible, per-operative cholangiography is a safe procedure concerning pancreas. Furthermore, per-operative cholangiography is of vital importance to visualize stones or tumors within the biliary ducts, a prerequisite to make the primary operation successful.

Åke Lasson
Depts of Surgery and Surgical Pathophysiology
Malmö General Hospital
Sweden

